# Demographic characteristics associated with food allergy in a Nationwide Canadian Study

**DOI:** 10.1186/s13223-021-00572-z

**Published:** 2021-07-17

**Authors:** Ann E. Clarke, Susan J. Elliott, Yvan St. Pierre, Lianne Soller, Sebastien La Vieille, Moshe Ben-Shoshan

**Affiliations:** 1 Division of Rheumatology, Cumming School of Medicine, University of Calgary, Calgary, AB Canada; 2 Department of Geography and Environmental Management, University of Waterloo, Waterloo, ON Canada; 3 Division of Clinical Epidemiology, Department of Medicine, McGill University Health Centre, Montreal, QC Canada; 4 Division of Allergy and Immunology, Department of Pediatrics, University of British Columbia, Vancouver, BC Canada; 5 Bureau of Chemical Safety, Health Canada, Ottawa, ON Canada; 6 Food Science Department, Faculty of Agricultural and Nutrition Sciences, Laval University, Quebec City, QC Canada; 7 Division of Allergy and Clinical Immunology, Department of Pediatrics, Montreal Children’s Hospital, McGill University Health Centre, Montreal, QC Canada

**Keywords:** Food allergy, Race/ethnicity, Sociodemographic, Multivariate analysis, Epidemiology

## Abstract

**Introduction:**

We conducted a nationwide Canadian telephone survey on food allergy prevalence between February 2016 and January 2017, targeting vulnerable populations (New, Indigenous, and lower-income Canadians).

**Objective:**

To examine the independent effect of demographic characteristics on food allergy.

**Methods:**

Canadian households with vulnerable populations were targeted using Canadian Census data and the household respondent reported whether each household member had a *perceived* (self-reported) or *probable* (self-report of a convincing history or physician diagnosis) food allergy. The association between *perceived* and *probable* food allergy and demographic characteristics was assessed through weighted multivariable random effects logistic regressions.

**Results:**

Children, females, Canadian-born participants, adults with post-secondary education, and those residing in smaller households were more likely to report *perceived* or *probable* food allergy. Although immigrant parents self-reported less food allergy, Canadian-born children of Southeast/East Asian immigrant versus other immigrant or Canadian-born parents reported more food allergy.

**Conclusion:**

We have demonstrated clear associations between demographic characteristics and food allergy, which may provide important clues to the environmental determinants of food allergy.

**Supplementary Information:**

The online version contains supplementary material available at 10.1186/s13223-021-00572-z.

To the editor,

## Introduction

We conducted a nationwide Canadian telephone survey on food allergy (FA) prevalence between February 2016 and January 2017 (SPAACE [**S**urveying **P**revalence of Food **A**llergy in **A**ll **C**anadian **E**nvironments] to SPAACE [S2S] [1]), targeting vulnerable populations (New, Indigenous, and lower-income Canadians) using 2006 Canadian Census data (Additional file 1). We compared prevalence between vulnerable and non-vulnerable populations [2] and showed (in univariable analysis) that overall prevalence was lower in immigrants and adults without post-secondary education. We now examine the independent effect of other demographic characteristics (age, sex, race/ethnicity, and household size), as well as immigrant status, education, and household income on presence of FA in multivariable analyses.

## Methods

Through the use of 2006 Canadian Census data, the census tracts (CT) from within the census metropolitan areas [3] that contained the highest proportion of households with New, Indigenous, and lower-income Canadian were selected and converted into postal codes using the 2006 Statistics Canada postal code conversion file. A random sample of household telephone numbers with accompanying mailing addresses was then selected from these postal codes by Info-Direct (a company maintaining household telephone directory listings in Canada: Cornerstone Info-Direct, Toronto, Ontario, Canada) and ASDE Survey Sampler (Gatineau, Quebec, Canada).

Due to the methodology for targeted sampling of the vulnerable populations, the smaller Canadian provinces (Newfoundland and Labrador, Prince Edward Island, Nova Scotia, and New Brunswick) as well as the Canadian territories (Northwest, Yukon, and Nunavut) were either excluded or not proportionately represented. Although our primary research objective was to ensure adequate representation of the vulnerable populations, we also wanted to provide nationwide prevalence estimates. Hence, we sampled households from additional regions from the four smaller provinces that contained the highest proportion of the vulnerable populations in these provinces although the percentage was lower than in the larger provinces. In the territories, a sample of households was obtained from the entire region.

The eligible adult household respondent (Additional file 1) completed the Food Allergy Prevalence Questionnaire (FAPQ) [1, 4, 5] in English or French, providing the following for each household member: allergy to peanut, tree nut, fish, shellfish, sesame, milk, egg, wheat, soy, or other foods, details on the most severe reaction for the nine allergens (symptoms, interval between exposure and symptoms, mode of contact, and if the allergy was physician-diagnosed), age, sex, race/ethnicity, birthplace, years in Canada (for non-Canadian-born), education (if ≥ 18 years), and household income. Food allergy was defined as *perceived* or *probable*. An individual was defined as having a *perceived* FA if they were reported by the adult household respondent to have any FA; an individual was defined as having a *probable* FA (Additional file 1) if the household respondent reported symptoms/signs compatible with a convincing history and/or physician diagnosis of a peanut, tree nut, fish, shellfish, sesame, milk, egg, wheat, and/or soy allergy [1, 2, 5].

The FAPQ included the following race/ethnicity options: South Asian (e.g. East Indian, Pakistani, Sri Lankan), Southeast Asian (e.g. Cambodian, Filipino, Indonesian, Laotian, Vietnamese), East Asian (i.e., Chinese, Japanese, Korean), Black, Indigenous (self-identified with First Nations, Metis, or Inuit), Arab, Latin American, West Asian (e.g., Afghan, Iranian, Iraqi), white, or other. In the analysis, race/ethnicity was stratified as South Asian, Southeast/East Asian, Black, Indigenous, white, or other (Arab, Latin American, West Asian, other, multiple, and unknown race/ethnicity).

The association between: (1) any *perceived* and (2) any *probable* FA and multiple demographic characteristics was assessed through weighted univariable and multivariable random effects logistic regressions, which allowed for correlated observations within households. For both outcomes, the most informative multivariable model was specified by selecting those demographic predictors for which an association remained statistically significant at the 95% confidence level, after eliminating all others, starting with the least likely to be associated with the outcome (backward stepwise selection). The Bonferroni method was also applied to assess the robustness of the associations. The association between each individual *perceived* and *probable* FA and demographic characteristics was examined using the same multivariable model selection process as for any *perceived* and *probable* FA. In addition, the influence of parental birthplace on any *perceived* and *probable* FA in Canadian-born children was assessed through the same multivariable model selection process. In the first regression, which examined the association between any *perceived* and *probable* FA and parental birthplace, the sample was restricted to include only parents with at least one Canadian-born child. In the second regression, which examined the association between any *perceived* and *probable* FA and parental birthplace, the sample included only Canadian-born children. In addition to the predictors considered in the model for *perceived* and *probable* FA which involved the full sample, the following predictors were also considered in the models with the restricted samples: South Asian-born, Southeast/East Asian-born, and non-Asian-born immigrant parent versus Canadian-born parent.

The Research Ethics Boards of the Universities of Calgary and Waterloo approved the study.

## Results

Of the 19,286 households targeted, 5874 of 11,592 eligible households completed the FAPQ, representing a 50.7% household response rate. Of 15,322 individuals participating, 14,818 provided sufficient information to be included in this analysis. Our sample consisted of 20.0% children, 48.4% of non-white race/ethnicity, 33.4% immigrants, 51.9% of adults without post-secondary education, and 22.7% resided in lower-income households (Table 1). Based on 2016 Canadian Census data [6], 20.0% of the general Canadian population were children, 27.2% were of non-white race/ethnicity, 21.9% were immigrants, 44.8% of adults did not have post-secondary education, and 12.8% resided in lower-income households. Given our targeted sampling strategy, the percentage of those of non-white race/ethnicity, immigrants, adults without post-secondary education, and residing in lower-income households exceeded the Canadian population.

**Table 1:** Demographic characteristics among full sample and those without and with *perceived* and *probable*^d^ food allergy

Variable	Full Sample (n = 14,818)	Frequency^e^, n (%)					
		Perceived			Probable		
		Without any *Perceived* FA (n = 13,497)	With Any *Perceived* FA (n = 1321)	Difference % (95% CI)	Without any *Probable* FA (n = 14,038)	With any of 9 *Probable* FA (n = 780)	Difference % (95% CI)
Age group, years							
0–17	2970 (20.0)	2750 (20.4)	220 (16.7)	**3.7 (1.6, 5.8)**	2816 (20.1)	154 (19.7)	0.3 (− 2.6, 3.2)
18–44	4142 (28.0)	3763 (27.9)	379 (28.7)	− 0.8 (− 3.4, 1.7)	3932 (28.0)	210 (26.9)	1.1 (− 2.1, 4.3)
≥ 45	7706 (52.0)	6984 (51.7)	722 (54.7)	− **2.9 (**− **5.7, **− **0.1)**	7290 (51.9)	416 (53.3)	− 1.4 (− 5.0, 2.2)
Female	7357 (49.6)	6575 (48.7)	782 (59.2)	− **10.5 (**− **13.3, **− **7.7)**	6893 (49.1)	464 (59.5)	− **10.4 (**− **13.9, **− **6.8)**
Race/ethnicity^a^							
South Asian	1072 (7.2)	1008 (7.5)	64 (4.8)	**2.6 (2.0, 3.3)**	1035 (7.4)	37 (4.7)	**2.6 (1.8, 3.4)**
Southeast/East Asian	1776 12.0)	1648 (12.2)	128 (9.7)	**2.5 (1.7, 3.4)**	1706 (12.2)	70 (9.0)	**3.2 (2.1, 4.2)**
Black	988 (6.7)	922 (6.8)	66 (5.0)	**1.8 (0.6, 3.1)**	941 (6.7)	47 (6.0)	0.7 (− 1.0, 2.4)
Indigenous	1788 (12.1)	1643 (12.2)	145 (11.0)	1.2 (− 0.6, 3.0)	1709 (12.2)	79 (10.1)	2 (− 0.1, 4.2)
White	7647 (51.6)	6849 (50.7)	798 (60.4)	− **9.7 (**− **12.4, **− **6.9)**	7174 (51.1)	473 (60.6)	− **9.5 (**− **13.1, **− **6.0)**
Other	1547 (10.4)	1427 (10.6)	120 (9.1)	1.5 (− 0.1, 3.1)	1473 (10.5)	74 (9.5)	1.0 (− 1.1, 3.1)
Immigration status							
New Canadians, immigrated < 10 years prior	1554 (10.5)	1481 (11.0)	73 (5.5)	**5.4 (4.1, 6.8)**	1514 (10.8)	40 (5.1)	**5.7 (4.0, 7.3)**
Immigrated ≥ 10 years prior	3389 (22.9)	3136 (23.2)	253 (19.2)	**4.1 (1.8, 6.3)**	3256 (23.2)	133 (17.1)	**6.1 (3.4, 8.9)**
Canadian-born	9875 (66.6)	8880 (65.8)	995 (75.3)	− **9.5 (**− **12, **− **7.1)**	9268 (66.0)	607 (77.8)	− **11.8 (**− **14.8, **− **8.8)**
Adults with post-secondary education^b^	5696 (48.1)	5119 (47.6)	577 (52.4)	− **4.8 (**− **7.9, **− **1.7)**	5357 (47.7)	339 (54.2)	− **6.4 (**− **10.4, **− **2.4)**
Household income							
Lower-income^c^	3362 (22.7)	3116 (23.1)	246 (18.6)	**4.5 (2.2, 6.7)**	3216 (22.9)	146 (18.7)	**4.2 (1.4, 7.0)**
Higher-income	9672 (65.3)	8754 (64.9)	918 (69.5)	− **4.6 (**− **7.2, **− **2.0)**	9135 (65.1)	537 (68.8)	− **3.8 (**− **7.1, **− **0.4)**
Missing income	1784 (12.0)	1627 (12.1)	157 (11.9)	0.2 (− 1.7, 2.0)	1687 (12.0)	97 (12.4)	− 0.4 (− 2.8, 2.0)
Household size, # of members (SD)	3.5 (1.8)	3.5 (1.8)	3.0 (1.6)	**0.5 (0.4. 0.6)**	3.5 (1.8)	3.1 (1.7)	**0.4 (0.3, 0.6)**

*FA* food allergy

Boldface cell indicate significant results

^a^Race/ethnicity options included: South Asian (e.g. East Indian, Pakistani, Sri Lankan), Southeast Asian (e.g. Cambodian, Filipino, Indonesian, Laotian, Vietnamese), East Asian (i.e., Chinese, Japanese, Korean), Black, Indigenous (self-identified with First Nations, Metis, or Inuit), Arab, Latin American, West Asian (e.g., Afghan, Iranian, Iraqi), white, or other. In the analysis, race/ethnicity was stratified as South Asian, Southeast/East Asian, Black, Indigenous, white, or other (Arab, Latin American, West Asian, other, multiple, and unknown race/ethnicity)

^b^Children < 18 years were not asked this information

^c^Lower-income Canadians were those whose self-reported before tax total household income was below the relevant low-income cut-off (LICO), as calculated yearly by Statistics Canada, for each of 7 household sizes and 5 community sizes. The LICO (before tax) is the income level at which families or unattached individuals spend on average 55% of before tax income on food, shelter, and clothing. Given we collected data on household income, household size, and postal code, we were able to ascertain if a household was below the LICO threshold [25]

^d^*Probable* food allergy was defined as any individual who was reported, by the household respondent, to have symptoms/signs compatible with a convincing history and/or a physician diagnosis of a peanut, tree nut, fish, shellfish, sesame, milk, egg, wheat, and/or soy allergy. Refer to Additional file 1 for definition of convincing history

^e^Except for household size where the mean (SD) number of household members is reported

In multivariable analyses, adults ≥ 45 years versus participants 0–44 years (OR 0.69, 95% confidence interval (CI) 0.56, 0.86), New Canadians versus Canadian-born (OR 0.51, 95% CI 0.38, 0.69), those immigrating to Canada ≥ 10 years prior versus Canadian-born (OR 0.75, 95% CI 0.62, 0.92) and those residing in larger households (OR 0.82, 95% CI 0.75, 0.90) were less likely to report any *perceived* FA (Table 2). Females (OR 1.49, 95% CI 1.27, 1.74) and adults with versus without post-secondary education (OR 1.20, 95% CI 1.02, 1.43) were more likely to report *perceived* FA, although this latter association did not remain significant when the Bonferroni correction was applied.

**Table 2:** Univariable and multivariable logistic regressions: demographic characteristics associated with *perceived* or *probable*^g^ food allergy, n = 14,818

Variable	Any *perceived* food allergy		Any of 9 *probable* food allergy	
	Univariable model Odds ratio (95% CI)	Multivariable model Odds ratio (95% CI)	Univariable model Odds ratio (95% CI)	Multivariable model Odds ratio (95% CI)
Age group (years)^a^				
0–17	0.91 (0.73, 1.12)	–	1.17 (0.91, 1.51)	**1.95 (1.38, 2.75)**
≥ 45	0.97 (0.83, 1.14)	**0.69 (0.56, 0.86)**	0.96 (0.78, 1.18)	–
Female	**1.46 (1.25, 1.70)**	**1.49 (1.27, 1.74)**	**1.47 (1.20. 1.80)**	**1.49 (1.22, 1.82)**
Race/ethnicity^b^				
South Asian	0.76 (0.55, 1.06)	–	0.77 (0.51. 1.17)	–
Southeast/East Asian	0.85 (0.65, 1.11)	–	0.86 (0.62, 1.21)	–
Black	0.84 (0.59, 1.19)	–	1.09 (0.72, 1.65)	–
Indigenous	1.02 (0.71, 1.46)	–	0.72 (0.42, 1.23)	–
Other	0.81 (0.61, 1.07)	–	0.84 (0.60, 1.18)	–
Immigration status^c^				
New Canadians, immigrated < 10 years prior	**0.45 (0.34, 0.61)**	**0.51 (0.38, 0.69)**	**0.39 (0.27, 0.57)**	**0.46 (0.30, 0.68)**
Immigrant ≥ 10 years	**0.71 (0.59, 0.84)**	**0.75 (0.62, 0.92)**	**0.60 (0.47, 0.76)**	**0.64 (0.49, 0.82)**
Post-secondary education^d^	**1.25 (1.06, 1.47)**	**1.20 (1.02, 1.43)**^**h**^	**1.33 (1.08, 1.64)**	**1.55 (1.23, 1.96)**
Household income				
Income missing	1.02 (0.80, 1.32)	–	1.01 (0.72, 1.40)	–
Low income^e^	0.94 (0.77, 1.14)	–	0.96 (0.75, 1.21)	–
Household size^f^	**0.85 (0.80, 0.92)**	**0.82 (0.75, 0.90)**	**0.88 (0.81, 0.95)**	**0.85 (0.77, 0.94)**

Empty cells indicate the variable was not included in the selected model

Boldface cells indicate significant results

^a^For univariable analysis, reference group is 18–44 years; For multivariable analysis, for *perceived* food allergy, reference group is 0–44 years and for *probable* food allergy, reference group is ≥ 18 years

^b^Race/ethnicity options included: South Asian (e.g. East Indian, Pakistani, Sri Lankan), Southeast Asian (e.g. Cambodian, Filipino, Indonesian, Laotian, Vietnamese), East Asian (i.e., Chinese, Japanese, Korean), Black, Indigenous (self-identified with First Nations, Metis, or Inuit), Arab, Latin American, West Asian (e.g., Afghan, Iranian, Iraqi), white, or other. In the analysis, race/ethnicity was stratified as South Asian, Southeast/East Asian, Black, Indigenous, white, or other (Arab, Latin American, West Asian, other, multiple, and unknown race/ethnicity). Reference group: White

^c^Reference group: Canadian-born

^d^Children < 18 years were not asked this information. Reference group: adults without post-secondary education

^e^Lower-income Canadians were those whose self-reported before tax total household income was below the relevant low-income cut-off (LICO), as calculated yearly by Statistics Canada, for each of 7 household sizes and 5 community sizes. The LICO (before tax) is the income level at which families or unattached individuals spend on average 55% of before tax income on food, shelter, and clothing. Given we collected data on household income, household size, and postal code, we were able to ascertain if a household was below the LICO threshold [25]. Reference group: Households that were not low income

^f^Household size is a continuous variable referring to number of members in the household

^g^*Probable* food allergy was defined as any individual who was reported, by the household respondent, to have symptoms/signs compatible with a convincing history and/or a physician diagnosis of a peanut, tree nut, fish, shellfish, sesame, milk, egg, wheat, and/or soy allergy. Refer to Additional file 1 for definition of convincing history

^h^Indicates that the OR is no longer significant when the Bonferroni correction is applied

New Canadians versus Canadian-born (OR 0.46, 95% CI 0.30, 0.68), those immigrating ≥ 10 years prior versus Canadian-born (OR 0.64, 95% CI 0.49, 0.82), and those residing in larger households (OR 0.85, 95% CI 0.77, 0.94) were less likely to report *probable* FA. Children versus adults aged ≥ 18 years (OR 1.95, 95% CI 1.38, 2.75), females (OR 1.49, 95% CI 1.22, 1.82) and adults with versus without post-secondary education (OR 1.55, 95% CI 1.23, 1.96) were more likely to report *probable* FA (Table 2).

Race/ethnicity and household income were not associated with either *perceived* or *probable* FA in multivariable models.

The association between demographic characteristics and the individual *perceived* and *probable* FA is reported in Additional file 1: Table S1A, B. Although many of the demographic characteristics associated with any *perceived* and *probable* FA were also associated with some of the individual FA, we also observed that race/ethnicity was associated with some individual FA. Those of Southeast/East Asian versus all other race/ethnicities were more likely to report *perceived* (OR 2.14, 95% CI 1.31, 3.50) and *probable* peanut allergy (OR 2.30, 95% CI 1.33, 3.97) and *perceived* shellfish allergy (OR 2.19, 95% CI 1.45, 3.30). Those of Southeast/East Asian versus those who were neither of Southeast/East Asian or Indigenous race/ethnicity were more likely to report *perceived* (OR 2.42, 95% CI 1.30, 4.53) and *probable* fish allergy (OR 4.06, 95% CI 1.87, 8.84) and *perceived* (OR 2.37, 95% CI 1.27, 4.41) and *probable* egg allergy (OR 2.41, 95% CI 1.26, 4.63). Those of South Asian versus non-Asian race/ethnicity (OR 0.25, 95% CI 0.08, 0.83) or of Southeast/East Asian versus non-Asian race/ethnicity (OR 0.32, 95% CI 0.11, 0.94) were less likely to report *perceived* wheat allergy. Those of Indigenous race/ethnicity versus those who were neither of Southeast/East Asian or Indigenous race/ethnicity were less likely to report *perceived* (OR 0.18, 95% CI 0.04, 0.81) and *probable* fish allergy (OR 0.19, 95% CI 0.04, 0.86) and *perceived* (OR 0.12, 95% CI 0.03, 0.55) and *probable* egg allergy (OR 0.12, 95% CI 0.03, 0.59). The associations of Asian race/ethnicities with *perceived* wheat allergy and of Indigenous race/ethnicity with *perceived* and *probable* fish allergy did not remain significant when the Bonferroni correction was applied.

When the sample was restricted to parents with at least one Canadian-born child, immigrant parents versus Canadian-born parents were less likely to report any *perceived* FA (South Asian immigrant: OR 0.24, 95% CI 0.11, 0.54; Southeast/East Asian immigrant: OR 0.52, 95% CI 0.28, 0.97); non-Asian immigrant: OR 0.46, 95% CI 0.28, 0.75). Similarly, immigrant parents versus Canadian-born parents were less likely to report any *probable* FA (South Asian immigrant: OR 0.23, 95% CI 0.08, 0.69; Southeast/East Asian immigrant: OR 0.32, 95% CI 0.12, 0.90; non-Asian immigrant: 0.42, 95% CI 0.22, 0.80) (Additional file 1: Table S2). When the Bonferroni correction was applied, the OR for Southeast/East Asian immigrant parents was no longer significant. However, Canadian-born children of Southeast/East Asian immigrant parents versus Canadian-born children of other immigrant parents or Canadian-born parents were more likely to report any *perceived* (OR 2.53, 95% CI 1.58, 4.04) and *probable* FA (OR 2.81, 95% CI 1.69, 4.69).

## Discussion

We have shown that while children, females, and adults with post-secondary education were more likely to report at least one *perceived* or *probable* FA and adults ≥ 45 years, immigrants, and those in larger households were less likely to report FA, Southeast/East Asian and Indigenous race/ethnicity were associated with *perceived* or *probable* allergy to specific foods. It is likely that the association between FA and higher education and Canadian birthplace is attributable to a variety of factors, including increased FA awareness and better healthcare access, which facilitates both self-reported and physician diagnosis of FA. Further, differing racial/ethnic, cultural, and socioeconomic influences on dietary practices and environmental exposures may also contribute [7–9]. Our observed association between larger household size and decreased FA supports the hygiene hypothesis, whereby increased exposure to siblings and the resultant increase in early childhood infections promotes a protective immune response [10].

Our findings that females and adults with higher education were more likely to report FA, whereas older adults were less likely to report FA are consistent with Gupta’s research [11]. Also in keeping with her work [12], we have shown in univariable analysis in a previous publication on this data that children in lower-income households were less likely to report any *perceived* FA (prevalence in lower income versus higher income households: 6.4% versus 9.2%, difference − 2.8%, 95% CI − 5.5%, − 0.1%) [2], whereas there was little association between household income and FA in adults. An Australian study also reported a higher prevalence of nut allergy in children of mothers with higher education and socio-economic index [13]. Our finding that certain FA (i.e., peanut, fish, shellfish, and egg) occurred more frequently in those of Asian race/ethnicity has also been reported by Gupta [11, 12] and others [14, 15]. A small Canadian study involving 151 respondents reported a higher prevalence of shellfish allergy in Historically Underrepresented (i.e., Indigenous, Asian, Black, and East Indian) versus white participants [14]. American studies have also reported a higher frequency of overall FA in Asian children [12, 15] and adults [11] although they have not examined South and Southeast/East Asians separately as we did. Food-induced anaphylaxis has been reported to occur more frequently in non-white children in both the United States [16] and the United Kingdom [17]. In contrast to the research by Gupta [11, 12] and others [15, 18–22], we did not observe an association of FA with Black race/ethnicity, potentially because of our small sample of this race/ethnicity (6.7%). Further, we observed that immigrants were less likely to report FA, whereas Gupta did not observe any association, potentially because her sample had a lower percentage of immigrants (8.4% versus 33.4% in ours) [11].

We observed lower odds of any *perceived* and *probable* FA in immigrant versus Canadian-born parents of Canadian-born children. Interestingly, for the the Southeast/East Asian immigrant parents, we observed higher odds of *perceived* and *probable* FA in their Canadian-born children versus Canadian-born children of other immigrant or Canadian-born parents. Consistent with our findings, an Australian study reported a higher frequency of peanut allergy in Australian-born infants of East Asian-born parents versus infants of Australian/British/European-born parents [23], while the prevalence of allergic disease was lower in Asian-born parents. A second Australian study reported a higher frequency of nut allergy in Australian-born children of both South and East Asian-born mothers versus children of non-Asian mothers. However, children born in Asia who migrated to Australia versus Australian-born children had a decreased risk of nut allergy [13]. Further, American-born children of immigrant versus American-born parents have been reported to have a higher odds of FA sensitization [24]. These observations suggest that early life environmental exposures, such as climate, dietary, and microbial, exert a differential effect on FA possibly depending on genetic background.

## Conclusion

Although our nationwide sampling frame precluded the use of food challenges to diagnosis FA and only included households with landlines and speaking either English or French, and nonresponse bias may have influenced our results, we have demonstrated clear associations between demographic characteristics and FA, potentially important clues to environmental determinants.

## Supplementary Information

**Additional file 1: Methods.** Additional methodological details on selection of study population, participant recruitment, telephone survey, and definition of probable food allergy. **Table S1A.** Multivariable logistic regression models: demographic characteristics associated with individual perceived food allergies. **Table S1B.** Multivariable logistic regression models: demographic characteristics associated with individual probable food allergies.  **Table S2.** Multivariate logistic regression models: association of parental immigrant status and perceived and probable food allergy.

## Data Availability

The data are not publicly available due to privacy or ethical restrictions.
