# Supplementary material for: Demographic characteristics associated with food allergy in a Nationwide Canadian Study
Source: Allergy Asthma Clin Immunol. 2021 Jul 17;17:72. doi: 10.1186/s13223-021-00572-z (PMC8285771; doi:10.1186/s13223-021-00572-z)
Supplement: Supplementary file 1 — Additional file 1: Methods. Additional methodological details on selection of study population, participant recruitment, telephone survey, and definition of probable food allergy. Table S1A. Multivariable logistic regression models: demographic characteristics associated with individual perceived food allergies. Table S1B. Multivariable logistic regression models: demographic characteristics associated with individual probable food allergies. Table S2. Multivariate logistic regression models: association of parental immigrant status and perceived and probable food allergy. [file 13223_2021_572_MOESM1_ESM.docx]

**ADDITIONAL FILE 1**

**METHODS**

**S2S: Surveying Prevalence of Food Allergy in All Canadian Environments (SPAACE) to SPAACE**

**Selection of study population**

New Canadians were those who reported immigrating to Canada within 10 years of completion of the telephone survey. Indigenous Canadians self-identified with First Nations, Metis, or Inuit. Lower-income Canadians were those whose self-reported before tax total household income was below the relevant low-income cut-off (LICO), as calculated yearly by Statistics Canada, for each of 7 household sizes and 5 community sizes. The LICO (before tax) is the income level at which families or unattached individuals spend on average 55% of before tax income on food, shelter, and clothing. Given we collected data on household income, household size, and postal code, we were able to ascertain if a household was below the LICO threshold.^25^

**Participant recruitment**

The S2S survey was conducted by the Survey Research Centre at the University of Waterloo, Ontario, Canada. All households, with the exception of Nunavut and some in the Northwest Territories where household addresses were unavailable, were mailed an information letter regarding the survey. As an unconditional incentive, a $5 coupon for a major Canadian restaurant chain was enclosed with the letter. When household addresses were unavailable, the study was advertised through a public service announcement and a $5 compensation was mailed to these households after completion of the survey and provision of their address.

**Telephone survey**

Approximately two weeks after mailing the information letter, households were contacted and invited to complete the Food Allergy Prevalence Questionnaire (FAPQ).^1,4,5^ A maximum of 15 attempts to contact the household was made during both weekdays and weekends. Respondents were eligible if they were 18 years or older, were living in the household, appeared to have no cognitive or hearing difficulties, could respond in either of Canada’s official languages (English or French), and were capable of answering questions about the FA of all household members.

**DEFINITION OF PROBABLE FOOD ALLERGY**

*Probable* food allergy was defined as any individual who was reported, by the household respondent, to have symptoms/signs compatible with a **convincing history** and/or a physician diagnosis of a peanut, tree nut, fish, shellfish, sesame, milk, egg, wheat, and/or soy allergy.

**Convincing history**

A history was considered convincing if the individual had experienced at least two **mild** symptoms or one **moderate** or one **severe** symptom after ingestion or contact (or inhalation for fish, shellfish, egg, or soy) within two hours after exposure to the food.^1,5^

**Mild** symptoms included: pruritis, urticaria, flushing or rhinoconjunctivitis.

**Moderate** symptoms included: angioedema, throat tightness, gastrointestinal symptoms, or breathing difficulties (other than wheeze).

**Severe** symptoms included: wheeze, cyanosis, or circulatory collapse.

**Excluding those with lactose intolerance or celiac disease from *probable* milk and wheat estimates:**

To ensure that those who had lactose intolerance or celiac disease were not erroneously considered to have milk or wheat allergy, those who reported either of these conditions or symptoms limited to the gastrointestinal tract or intermittent ability to tolerate dairy or wheat products were excluded from our estimates of *probable* milk or wheat allergy.

**Additional File 1. Table 1A. Multivariable Logistic Regression Models: Demographic Characteristics Associated with Individual *Perceived* Food Allergies, Odds Ratio (95% CI), n=14,818**

| **Variable** | **Peanut** | **Tree nut** | **Fish** | **Shellfish** | **Sesame** | **Milk** | **Egg** | **Wheat** | **Soy** | **Other** |
| --- | --- | --- | --- | --- | --- | --- | --- | --- | --- | --- |
| **Age group**^1^ |  |  |  |  |  |  |  |  |  |  |
| 0-17 yrs | 2.07  (1.31, 3.30) | 2.14  (1.33, 3.43) | 1.80*  (1.02, 3.17) | - | 3.68  (1.19, 11.30) | - | 2.71  (1.63, 4.53) | - | - | 0.45  (0.29, 0.70) |
| ≥45 yrs | 0.40  (0.24, 0.67) | - | - | - | - | - | - | - | - | 0.60  (0.43, 0.84) |
| **Female** | - | 1.58*  (1.09, 2.28) | - | - | - | 1.82  (1.36, 2.44) | - | 1.82*  (1.10, 3.00) | 2.78  (1.30, 5.92) | 1.63  (1.27, 2.10) |
| **Race/**  **Ethnicity^2^** |  |  |  |  |  |  |  |  |  |  |
| South Asian | - | - | - | - | - | - | - | 0.25*  (0.08, 0.83) | - | - |
| Southeast /East Asian | 2.14  (1.31, 3.50) | - | 2.42  (1.30, 4.53) | 2.19  (1.45, 3.30) | - | - | 2.37  (1.27, 4.41) | 0.32*  (0.11, 0.94) | - | - |
| Indigenous | - | - | 0.18*  (0.04, 0.81) | - |  | - | 0.12  (0.03, 0.55) | - | - | - |
| **Immigration status**^3^ |  |  |  |  |  |  |  |  |  |  |
| New Canadians, immigrated < 10 yrs prior | 0.13  (0.05, 0.31) | 0.11  (0.04, 0.34) | - | - | - | 0.42  (0.23, 0.78) | 0.16  (0.06, 0.40) | - | - | 0.39  (0.23, 0.66) |
| Immigrant ≥ 10 yrs |  | 0.33  (0.20, 0.55) | - | - | - | 0.66*  (0.46, 0.93) | 0.45*  (0.21, 0.94) | 1.86*  (1.13, 3.07) | - | 0.70*  (0.51, 0.95) |
| **Post-secondary education**^4^ | - | 1.92  (1.25, 2.95) | - | - | - | - | - | - | - | - |
| **Household size**^5^ | - | - | - | 0.80  (0.71, 0.89) | 0.62  (0.44, 0.88) | 0.75  (0.67, 0.84) | - | 0.74  (0.60, 0.91) | 0.69  (0.54, 0.87) | 0.84*  (0.73, 0.96) |

^1^ For peanut and other food allergies, reference group is 18 – 44 years; for tree nut, fish, sesame, and egg allergy, reference group is all adults.

^2^ Race/ethnicity options included: South Asian (e.g. East Indian, Pakistani, Sri Lankan), Southeast Asian (e.g. Cambodian, Filipino, Indonesian, Laotian, Vietnamese), East Asian (i.e., Chinese, Japanese, Korean), Black, Indigenous (self-identified with First Nations, Metis, or Inuit), Arab, Latin American, West Asian (e.g., Afghan, Iranian, Iraqi), white, or other. In the analysis, race/ethnicity was stratified as South Asian, Southeast/East Asian, Black, Indigenous, white, or other (Arab, Latin American, West Asian, other, multiple, and unknown race/ethnicity).

For peanut and shellfish allergy, reference group is non-Southeast/East Asian; For fish and egg allergy, reference group is all who are neither of Southeast/East Asian or Indigenous race/ethnicity; For wheat allergy, reference group is non-Asian.

^3^ For peanut allergy, reference group is immigrants ≥ 10 years or Canadian-born; For tree nut, milk, egg, and other food allergies, reference group is Canadian-born; For wheat allergy, reference group is New Canadians or Canadian-born.

^4^ Children < 18 years were not asked this information. Reference group: adults without post-secondary education

^5^ Household size is a continuous variable referring to number of members in the household.

* Indicates that the OR would not be significant when the Bonferroni correction is applied.

Empty cells indicate the variable was not included in the selected model.

**Additional File 1. Table 1B.** **Multivariable Logistic Regression Models: Demographic Characteristics Associated with Individual *Probable***† **Food Allergies, Odds Ratio (95% CI), n=14,818**

| **Variable** | **Peanut** | **Tree nut** | | **Fish** | **Shellfish** | | | **Sesame** | **Milk** | | | **Egg** | | | **Wheat** | | **Soy** | | |
| --- | --- | --- | --- | --- | --- | --- | --- | --- | --- | --- | --- | --- | --- | --- | --- | --- | --- | --- | --- |
| **Age group**^1^ |  |  | |  |  | | |  |  | | |  | | |  | |  | | |
| 0-17 yrs | 1.83*  (1.14, 2.95) | 2.04  (1.22, 3.41) | - | | | - | - | | | 2.17  (1.20, 3.94) | 2.61  (1.53, 4.44) | | | - | | | | - | |
| ≥ 45 yrs | 0.32  (0.19, 0.57) | - | - | | | - | - | | | - | - | | | - | | | | - | |
| **Female** | - | - | - | | | 1.59  (1.06, 2.38) | - | | | 2.10  (1.38, 3.20) | - | | | 1.93*  (1.01, 3.67) | | | | 2.59*  (1.11, 6.07) | |
| **Race/Ethnicity^2^** |  |  |  | | |  |  | | |  |  | | |  | | | |  | |
| Southeast/East Asian | 2.30  (1.33, 3.97) | - | 4.06  (1.87, 8.84) | | | - | - | | | - | 2.41  (1.26, 4.63) | | | - | | | | - | |
| Indigenous | - | - | 0.19*  (0.04, 0.86) | | | - | - | | | - | 0.12  (0.03, 0.59) | | | - | | | | - | |
| **Immigration status**^3^ |  |  |  | | |  |  | | |  |  | | |  | | | |  | |
| New Canadians, immigrated < 10 yrs prior | 0.11  (0.04, 0.29) | 0.06  (0.01, 0.30) | 0.26*  (0.08, 0.82) | | | - | - | | | - | | | 0.12  (0.04, 0.35) | | | - | | | - |
| Immigrant ≥ 10 yrs | 0.43*  (0.22, 0.82) | 0.27  (0.15, 0.48) | 0.37*  (0.16, 0.85) | | | - | - | | | - | | | 0.26  (0.10, 0.71) | | | 1.85*  (1.04, 3.28) | | | - |
| All immigrants | - | - | - | | | - | 0.21  (0.05, 0.95) | | | - | | | - | | | - | | | - |
| **Post-secondary education**^4^ | - | 2.08  (1.31, 3.31) | - | | | - | - | | | - | - | | | - | | | | - | |
| **Household size**^5^ | - | - | - | | | 0.83  (0.73, 0.95) | - | | | 0.63  (0.52, 0.75) | - | | | 0.66  (0.52, 0.83) | | | | 0.73  (0.57, 0.93) | |

^1^ For peanut allergy, reference group is 18 – 44 years; for tree nut, milk, and egg allergy, reference groups is all adults.

^2^ Race/ethnicity options included: South Asian (e.g. East Indian, Pakistani, Sri Lankan), Southeast Asian (e.g. Cambodian, Filipino, Indonesian, Laotian, Vietnamese), East Asian (i.e., Chinese, Japanese, Korean), Black, Indigenous (self-identified with First Nations, Metis, or Inuit), Arab, Latin American, West Asian (e.g., Afghan, Iranian, Iraqi), white, or other. In the analysis, race/ethnicity was stratified as South Asian, Southeast/East Asian, Black, Indigenous, white, or other (Arab, Latin American, West Asian, other, multiple, and unknown race/ethnicity).

For peanut allergy, reference group is non-Southeast/East Asian; For fish and egg allergy, reference group is all who are neither of Southeast/East Asian or Indigenous race/ethnicity.

^3^ For peanut, tree nut, fish, sesame, and egg allergy, reference group is Canadian-born; for wheat, reference group is New Canadians or Canadian-born.

^4^ Children < 18 years were not asked this information. Reference group: adults without post-secondary education

^5^ Household size is a continuous variable referring to number of members in the household.

† *Probable* food allergy was defined as any individual who was reported, by the household respondent, to have symptoms/signs compatible with a convincing history and/or a physician diagnosis of a peanut, tree nut, fish, shellfish, sesame, milk, egg, wheat, and/or soy allergy. Refer to Additional File 1 for definition of convincing history.

* Indicates that the OR would not be significant when the Bonferroni correction is applied.

Empty cells indicate the variable was not included in the selected model.

**Additional File 1. Table 2. Multivariable Logistic Regression Models: Association of Parental Immigrant Status and *Perceived* or *Probable***† **Food Allergy, Odds Ratio (95% CI)**

|  | **Any *perceived* food allergy** | **Any of 9 *probable* food allergies** |
| --- | --- | --- |
|  | **OR (95% CI)** | **OR (95% CI)** |
| **Parents with Canadian-born children** | |  |
| n=3218 |  |  |
| South Asian immigrant^1^ | 0.24 (0.11, 0.54) | 0.23 (0.08, 0.69) |
| Southeast/East Asian immigrant^1^ | 0.52 (0.28, 0.97)* | 0.32 (0.12, 0.90)* |
| Non-Asian immigrant^1^ | 0.46 (0.28, 0.75) | 0.42 (0.22, 0.80) |
| Post-secondary education | - | 2.03 (1.19, 3.47) |
| **All Canadian-born children** |  |  |
| n=2498 |  |  |
| At least 1 Southeast/East Asian immigrant parent^2^ | 2.53 (1.58, 4.04) | 2.81 (1.69, 4.69) |
| Household size*^3^* | 0.79 (0.66, 0.95) | 0.78 (0.64, 0.94) |

^1^ Reference group is Canadian-born parent

^2^ Reference group is children with immigrant parents other than Southeast/East Asian or Canadian-born parents

^3^ Household size is a continuous variable referring to number of members in the household.

† *Probable* food allergy was defined as any individual who was reported, by the household respondent, to have symptoms/signs compatible with a convincing history and/or a physician diagnosis of a peanut, tree nut, fish, shellfish, sesame, milk, egg, wheat, and/or soy allergy. Refer to Additional File 1 for definition of convincing history.

* Indicates that the OR would not be significant when the Bonferroni correction is applied.

Empty cells indicate the variable was not included in the selected model.
